# King George's Bed

**Published:** 1915-11-13

**Authors:** 


*To the Editor of* THE HOSPITAL.

### King George's Bed.

*To the Editor of* THE HOSPITAL.

SIR,—On the arrival of the King at Buckingham Palace, after meeting with the accident in France, caused by his horse rearing at the applause, his bed at the Palace was pronounced unsuitable on account of it not being fitted with the Staples spiral mattress—a form of mattress which is made of spiral springs, each spring moving upwardly and independently, and thus supporting the body equally at all points. This is, of course, in direct contradistinction to the usual form of hospital and other wire mattresses, which, being suspended from the edges only, tend to sag into a hollow in the centre. Accordingly a new bedstead was immediately ordered, it being stipulated that it must be fitted with a Staples mattress. This order was received from Buckingham Palace by Messrs. Heal and Son, Tottenham Court Road, together with one for their best quality special hair mattresses.—Yours faithfully,
STAPLES AND CO.
Chitty Street Works, November 3, 1915.

